# Implementation of Best Practices in Obesity Prevention in Child Care Facilities: The Arizona Empower Program, 2013–2015

**DOI:** 10.5888/pcd14.160451

**Published:** 2017-09-07

**Authors:** Jillian Papa, Joan Agostinelli, Gertrudes Rodriguez, Deborah Robinson

**Affiliations:** 1 Arizona Department of Health Services, Phoenix, Arizona

## Abstract

**Introduction:**

Obesity is a major health concern in every US age group. Approximately one in 4 children in Arizona’s Special Supplemental Nutrition Program for Women, Infants, and Children is overweight or obese. The Arizona Department of Health Services developed the Empower program to promote healthy environments in licensed child care facilities. The program consists of 10 standards, including one standard for each of these 5 areas: physical activity and screen time, breastfeeding, fruit juice and water, family-style meals, and staff training. The objective of this evaluation was to determine the level of implementation of these 5 Empower standards.

**Methods:**

A self-assessment survey was completed from July 2013 through June 2015 by 1,850 facilities to evaluate the level of implementation of 5 Empower standards. We calculated the percentage of facilities that reported the degree to which they implemented each standard and identified common themes in comments recorded in the survey.

**Results:**

All facilities reported either full or partial implementation of the 5 standards. Of 1,678 facilities, 21.7% (n = 364) reported full implementation of all standards, and 78.3% (n = 1,314) reported at least partial implementation. Staff training, which has only one component, had the highest level of implementation: 77.4% (n = 1,299) reported full implementation. Only 44.0% (n = 738) reported full implementation of the standard on a breastfeeding-friendly environment.

**Conclusion:**

Arizona child care facilities have begun to implement the Empower program, but facilities will need more education, technical assistance, and support in some areas to fully implement the program.

## Introduction

The US Surgeon General recognizes obesity as a major health concern (1). Obese children as young as 2 to 5 years are more likely than children who are not obese at that age to become obese adults (2). Approximately one in 4 children aged 2 through 4 years enrolled in Arizona’s Special Supplemental Nutrition Program for Women, Infants, and Children (WIC) is overweight or obese (3).

Approximately 80% of preschool-aged children spend as much as 40 hours per week in nonparental care (4). National recommendations have established the early care and education setting as an important opportunity for obesity intervention (5). Evaluations of obesity prevention interventions in child care facilities show improvements in children’s mealtime behaviors, dietary preferences, and levels of physical activity (4).

Several prominent authorities collaboratively published national standards for best practices in 2010 (6). The Empower program, based on these national standards, was implemented in 2010 to promote healthy environments for children in Arizona’s licensed child care facilities. Participating facilities receive discounted licensing fees for their agreement to follow the program’s 10 standards (Box).

**Box. Ten Empower Standards to Improve Health in Licensed Child Care Facilities in Arizona^a,b^**

**Standard 1. Provide at least 60 minutes of daily physical activity, and do not allow more than 3 hours of screen time per week.**

Standard 2. Practice sun safety.

**Standard 3. Provide a breastfeeding-friendly environment.**

Standard 4. Determine whether site is eligible for the US Department of Agriculture Child and Adult Care Food Program.

**Standard 5. Limit serving fruit juice.**

**Standard 6. Serve meals family style.**

Standard 7. Provide monthly oral health care education or implement a tooth-brushing program.

**Standard 8. Ensure that staff members receive 3 hours of training annually on Empower topics.**

Standard 9. Make Arizona Smokers’ Helpline (ASHLine) education materials available at all times.

Standard 10. Maintain a smoke-free campus.

^a^ Data source: Arizona Department of Health Services Bureau of Nutrition and Physical Activity (7).

^b^ Implementation of standards in boldface were evaluated in this study.

The objective of this evaluation was to determine the level of implementation of the 5 Empower standards that relate to obesity prevention: the standards on physical activity and screen time, breastfeeding, fruit juice and water, and family-style meals, and the standard on staff training. We hypothesized that none of the standards would have been implemented across all participating facilities.

## Methods

We designed a self-assessment survey to collect data from licensed child care facilities enrolled in Arizona’s Empower program between July 1, 2013, and June 30, 2015.

Child care facilities volunteer to implement 10 Empower standards. As part of the program, participating facilities receive the Empower guidebook (7), a reference manual on early care and education best practices and national recommendations (6,8), and documents on state rules and regulations (9,10). The guidebook defines key terms related to standards and provides age-specific guidance and adaptations for special circumstances. Examples of policies and materials were also provided for staff education, family engagement, and marketing to assist with implementation. 

The Arizona Department of Health Services (ADHS) inspects all types of licensed child care facilities at least once annually. The data for this study were collected during the first inspection completed for each participating facility during the 2-year study period. At least one assessment was completed by 1,862 facilities. Data for 12 facilities were excluded because the provider identification number did not match information in the licensing database, which we used to verify the identity of each facility and its enrollment capacity. Our sample consisted of 1,850 facilities serving 182,602 children.

Surveyors from the ADHS Bureau of Child Care Licensing collected data from a facility staff member during their annual on-site inspections. In year 1, electronic tablets and paper surveys were used to collect data, which were then entered into an Excel (Microsoft Corp) form. Surveyors requested paper surveys in year 2; data from these surveys were also entered into an Excel form. These data were then compiled into an Empower database in Excel. Surveyors also recorded comments from facility staff.

The data collection tool was a self-administered survey that asked facilities to self-report their level of implementation on each component of each standard. Four of the 5 standards consist of more than one component: physical activity and screen time (10 components), breastfeeding (4 components), fruit juice and water (7 components), and family-style meals (6 components). The fifth standard, staff training, has only one component. Each component represents a discrete, observable aspect of the standard.

We conducted 3 levels of analysis: by component, by standard, and by facility. Proportions were calculated for each component, standard, and facility by using SPSS version 24 (IBM Corp). For the analysis by component, each component was examined for its level of implementation as either full, partial, or not at all. Missing values and responses of “don’t know” were combined into one category. Because the staff training standard has only one component, we did not analyze this standard by component.

For the analysis by standard, we categorized each standard into an overall rating across its components. A standard was categorized as fully implemented when a facility reported fully implementing all of the components of the standard. The standard was categorized as not at all being implemented when a facility reported implementation of all components of the standard as “not at all.” If a facility reported any other mix of ratings across components, the standard was categorized as partially implemented. Components with missing values and responses of “don’t know” were excluded from the analyses by standard. The analysis by standard consisted of 1,678 assessments.

For the analysis by facility, we calculated the percentage of facilities that implemented standards fully, partially, or not at all. A facility was classified as fully implemented if all standards were rated as fully implemented. Facilities that had implemented none of the standards were classified as “not at all” implemented. Any facility with any other combination of fully or partially implemented standards was classified as partially implemented. Components with missing values and responses of “don’t know” were excluded from the analyses by facility. The analysis by facility consisted of 1,678 assessments.

We conducted a content analysis of comments by using QDA Miner version 4.0 (Provalis Research). We identified themes and developed codes for analysis. The 1,850 assessments offered 283 comments; we excluded 77 comments because they were not relevant to the study. The remaining 206 comments were classified into one of 6 categories (general Empower program and the 5 standards) and then analyzed for themes. 

## Results

In the analysis by facility, all 1,678 facilities reported either full or partial implementation of all 5 standards. About one-fifth (21.7%; n = 364) of facilities reported full implementation of all standards, and 78.3% (n = 1,314) of facilities reported at least partial implementation. In the analysis by standard, the percentage of child care facilities reporting full implementation ranged from 44.0% (n = 738), for the breastfeeding-friendly environment, to 77.4% (n = 1,299) for staff training (Figure). In the analysis by component (Table), the level of full implementation ranged from 45.6% (provides breastfeeding information to families) to 97.9% (offers water as the first choice for thirst). Four standards had a component on providing information to families; these components had the lowest levels of implementation. We observed the highest levels of implementation for the standard on fruit juice and water.

**Figure:**
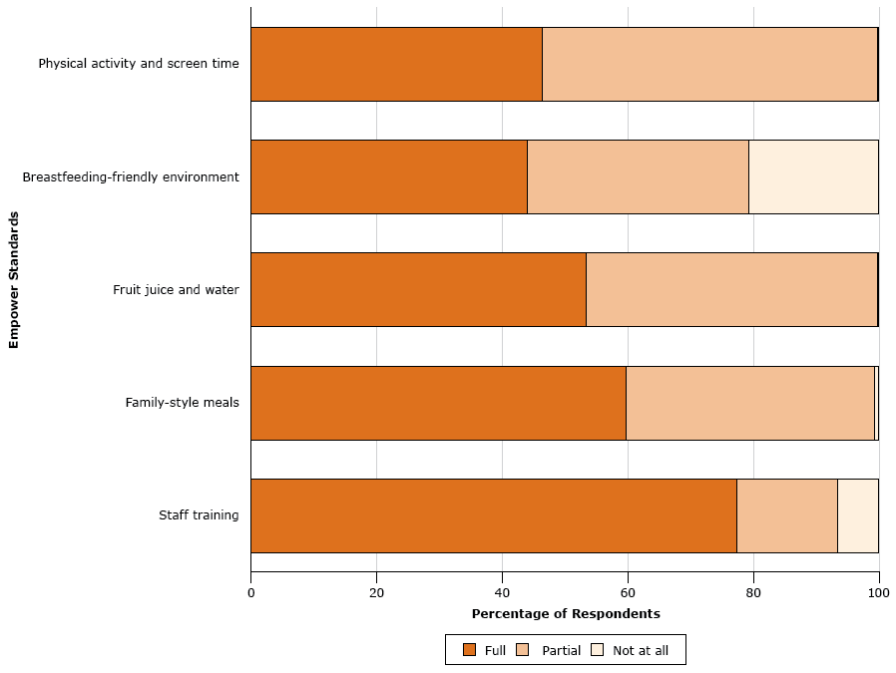
Level of implementation, by standard, of Arizona Licensed Child Care Facilities Empower standards reported by 1,678 child care facilities, July 2013–June 2015. Components with missing values and responses of “don’t know” were excluded from the analyses by standard. Four standards have multiple components; the staff training standard has only one component. **Table:** 

Standard	Percentage of Respondents		
	Full	Partial	Not at All
Physical activity and screen time	46.3	53.6	0.1
Breastfeeding-friendly environment	44.0	35.3	20.7
Fruit juice and water	53.3	46.6	0.1
Family-style meals	59.7	39.6	0.7
Staff training	77.4	16.1	6.5

**Table:** Results of Survey Among Licensed Child Care Facilities in Arizona (n = 1,850) on Level of Implementation of Selected Empower Standards, by Component, July 2013–June 2015^a^^,^ b

Component	Percentage of Respondents Indicating Level of Implementation			
	Fully Implemented	Partially Implemented	Not at All Implemented	Response of Don’t Know or Missing Data
**Standard 1: Physical activity and screen time**				
Provides at least 60 minutes of planned physical activity per day	89.1	9.7	0.3	0.9
Includes teacher-led activities	86.5	12.3	0.4	0.7
Includes free play opportunities	92.5	6.9	0.1	0.5
Includes outdoor physical activity	91.1	7.5	0.9	0.5
Includes moderate physical activity	87.4	11.5	0.4	0.8
Includes vigorous physical activity	67.2	26.7	4.3	1.8
Limits sedentary activity to no more than 60 minutes at a time (except nap time)	89.2	6.2	2.5	2.1
Limits screen time to 3 hours or less per week	88.5	4.3	5.9	1.3
Prohibits use of physical activity as punishment	89.9	1.5	7.9	0.7
Provides information on screen time to families	67.0	15.9	13.9	3.2
**Standard 3: Breastfeeding-friendly environment**				
Provides a place to breastfeed or express milk (not a bathroom)	62.3	4.8	27.8	5.1
Provides a refrigerator for milk storage	71.4	1.6	23.1	3.9
Displays breastfeeding promotion information	47.8	7.7	38.1	6.4
Provides breastfeeding information to families	45.6	10.5	37.5	6.4
**Standard 5: Fruit juice and water**				
Offers water throughout the day	98.6	0.6	0.3	0.5
Offers water as the first choice for thirst	97.9	1.2	0.4	0.5
Prohibits serving fruit juice more than twice per week for children aged 1 year or older	84.7	8.0	6.1	1.2
Prohibits serving more than 4 to 6 ounces of fruit juice at one time	88.7	5.6	4.1	1.7
Serves juice that is only 100% fruit juice with no added sugar	91.4	2.9	4.4	1.3
Serves fruit juice only at meal or snack times	88.5	4.9	5.4	1.2
Provides information on fruit juice to families	59.7	15.8	20.1	4.4
**Standard 6: Family-style meals**				
Serves meals family style	78.7	12.8	7.2	1.2
Uses child-friendly serving utensils	85.7	7.6	5.6	1.0
Requires staff participation in meal-time with children	91.2	5.8	2.2	0.9
Allows children to choose what and how much to eat	83.4	12.6	3.2	0.7
Prohibits using food as a punishment or reward	93.2	0.9	4.7	1.2
Provides information on healthy eating to families	78.4	13.6	6.3	1.7

a Percentages do not always sum to 100 because of rounding. All percentages are based on a denominator of 1,850.

b The standard on training is not included because it has no components.

Of the 206 relevant comments, 92.7% (n = 191) were from surveys completed during the first year of the 2-year study period. Seventy-five comments related to the Empower program in general; of these, 33 (44.0%) comments related to the process of developing policies; 35 (46.7%) comments related to being new to the program and not receiving, or only recently receiving, the guidebook; 3 (4.0%) comments related to changes in personnel; and 4 (5.3%) comments indicated that the respondent did not know if the facility had a policy. The other 139 relevant comments related to one of the 5 standards and are summarized below.

**Physical activity and screen time. **Nearly half (46.3%) of facilities reported fully implementing all 10 components of the standard on physical activity and screen time (Figure). Most reported fully implementing the component on providing free-play opportunities (92.5%) and outdoor activity (91.1%), and 67.2% reported fully implementing the component on providing vigorous physical activity. Providing information on screen time to families had the lowest level of implementation (67.0%). Of the 55 comments on physical activity and screen time, 11 (20.0%) comments related to the need to clarify language or confusion about components, and 14 (25.5%) comments related to facilities not being all-day centers. Some respondents asked about the definitions of words such as vigorous, moderate, sedentary, and screen time. Other comments related to a facility being a partial-day facility or not having an outdoor playground.

**Breastfeeding-friendly environment.** The breastfeeding-friendly environment standard had the lowest level of implementation. Only 44.0% of facilities reported full implementation, and 20.7% reported that they had not implemented any components. The lowest component scores for this standard were in providing information to families (45.6%) and displaying information (47.8%). Of the 76 comments related to breastfeeding-friendly environments, most (67.1%) indicated that the respondent’s facility did not provide care for infants and 16 (21.1%) indicated that the standard was not applicable.

**Fruit juice and water.** More than half (53.3%) of facilities reported fully implementing all of the components of the standard on fruit juice and water. Nearly all reported full implementation of 2 components: offering water throughout the day (98.6%) and offering water as the first choice for thirst (97.9%). Most (91.4%) facilities reported fully implementing the component for serving only 100% fruit juice. The component for providing information on fruit juice and water to families had the lowest level of implementation, with only 59.7% of facilities reporting full implementation. Of the 37 comments related to the standard on fruit juice and water, 27 (73.0%) indicated not serving fruit juice at all.

**Family-style meals.** More than half (59.7%) of facilities reported fully implementing all components of family-style meals. Most (93.2%) reported full implementation of the component for prohibiting the use of food as punishment or reward, and most (91.2%) required staff participation in meals. More than four-fifths fully implemented the components for using child-friendly serving utensils (85.7%) and allowing children to choose what and how much to eat (83.4%). The component of this standard with the lowest level of implementation was providing information to families (78.4%). Of the 35 comments related to family-style meals, 18 (51.4%) were about children bringing their own food from home. Three comments were about not serving food at all (8.6%).

**Staff training.** The staff training standard had the highest percentage (77.4%) of facilities reporting full implementation. We found 3 comments on staff training.

## Discussion

Approximately one in 5 licensed child care facilities in Arizona reported full implementation of all 10 standards of the Empower program; most facilities reported at least partially implementing them. Survey comments suggested that many facilities were not familiar with the Empower guidebook, especially the sections explaining key terms related to physical activity and offering guidance on partial days and indoor play. It appears that more training and technical assistance are needed on these topics. Future training and technical assistance will focus on educating facility staff members on these topics, which are fundamental in transforming the early care and education setting to support healthy behaviors (11).

A breastfeeding-friendly environment is another important area of focus for obesity prevention. Observational studies suggest that the first 2 years of life are an optimal time in which to prevent obesity by establishing positive eating behaviors (12). They also suggest that breastfeeding has a promising role in preventing childhood obesity (13,14). The Empower breastfeeding standard focuses on creating a breastfeeding-friendly environment (7), which applies to all child care facilities, even if they do not serve infants. This standard had the lowest rate of implementation, with comments indicating that many thought the standard did not apply to facilities that did not care for infants. These comments suggest that facilities will need additional training and technical assistance on this topic. Child care staff members may need a place to express and store breastmilk for their own breastfeeding children who are not at the facility. Alternatively, a mother of an older enrolled child may need a private lactation area to breastfeed a younger sibling. A breastfeeding-friendly environment should not be limited to facilities that enroll infants.

Arizona’s standard on fruit juice is more restrictive than the national recommendation to serve no more than 4 to 6 ounces of fruit juice per day to children aged one to 6 years (6). Arizona’s standard requires no more than 2 four-ounce servings of 100% fruit juice per week be given to children aged one to 6, unless it is appropriate for a child’s special health care need (7). Water and milk are the preferred beverages for meals and snacks (7). Nearly all facilities reported full implementation of components for offering water throughout the day and offering water as the first choice for thirst. Arizona’s revised statutes on fruit and fruit juice align with national recommendations; however, Empower standards exceed those requirements. Empower training in standards on accessible and abundant water is especially emphasized in Arizona because of the potential for dehydration in extreme temperatures. It is critically important to comply with this requirement in Arizona, where temperatures in the shade frequently measure more than 110°F.

The Institute of Medicine recommends that child care providers practice responsive feeding, which includes self-regulation of intake by infants and allowing toddlers and preschoolers to serve themselves from common bowls (family-style service) (15). This practice encourages children to eat according to their own hunger and fullness cues (16) and develop their hand–eye coordination skills. Additionally, the presence of an adult facilitates learning about nutrition and can lead to pleasant mealtimes (6). Implementation levels for serving meals family-style were relatively high among the facilities studied.

Parents and families are role models and strongly influence young children’s eating and activity environments (14,17). Communication between child care staff and parents is important to promote healthy weight in children (4). Each standard includes a component on providing information to the family, and we found that this component had the lowest level of implementation in each standard, indicating a need for specific, standardized educational materials for all facilities to display, disseminate, and have available for families. Anecdotal feedback from monthly meetings with the licensing surveyors indicated that child care staff were receptive to distributing and posting colorful educational materials. They suggested that providing a variety of educational information on a regular basis could help improve family engagement on Empower topics.

A strength of the study is the size of the sample. The ADHS Bureau of Nutrition and Physical Activity leveraged a unique partnership with the Bureau of Child Care Licensing to administer the assessments as part of its routine site inspections. By embedding the assessments into licensing processes, we were able to collect data throughout the state using fewer resources and less time than we would have used otherwise. The Bureau of Child Care Licensing surveyors also were able to offer education and technical assistance to encourage facilities to implement standards. Other states may want to explore this type of opportunity with their child care regulatory agencies to leverage similar efforts.

Our study had several limitations, including selection and response biases. That facilities volunteered to implement the 10 standards and participate in the program is a selection bias. Discounted licensing fees provided a monetary incentive to overstate levels of implementation. In addition, the self-assessment was part of a site inspection in which a reviewer had authority to sanction noncompliance of official rules. These factors exerted potentially strong external pressures toward favorable responses. The Empower survey had never been used before and has not been validated. It was a self-assessment, and there was no way to know how closely a respondent’s self-reported responses corresponded with their actual practices. The survey did not have an option for “does not apply” because standards were designed to apply to all settings. Although modifications of standards are permitted for the unique aspects of some sites, the survey did not account for these modifications. Finally, this evaluation was limited to ADHS-licensed child care facilities, and our findings might not generalize to other early care and education settings in or outside Arizona.

Despite these limitations, our results provide insights into an ongoing statewide effort to implement the Empower program in licensed child care facilities and provide baseline data against which future measures can be assessed. Low levels of implementation in providing information to families show a need to develop standard educational materials for families, while state requirements have led to high levels of implementation for the most stringent standards, such as fruit juice and water, suggesting that family engagement should be a focus of further study. Our findings will be used in Arizona to more effectively promote policy, system, and environmental changes in child care settings, which have the potential to improve the health of and reduce obesity rates among preschool-aged children in Arizona. The program is still in a capacity-building phase, and it is too early to assess its effect on childhood obesity rates.
